# Case of Strangulated Crural Hernia, Operated on According to the Method of Don Antonio Gimbernat; with Some Observations on the Treatment of Hernia

**Published:** 1810-05

**Authors:** John C. Warren


*Case of Strangulated Crural Hernia, operated on according to the Method of Don Antonio Gimbernat; with some Observations on the Treatment of Hernia; by* JOHN C. WARREN, M. D.

( Read before the Massachusetts Medical Society. )

THE operation for the relief of Strangulated Crural Hernia has been formidable to good surgeons, because not well understood. Mr. Pott speaks of it with apprehension; and the method of performing it proposed by Mr. Benjamin

---

Benjamin Bell seems so inadequate, that it must have been a difficult affair under his hands. An accurate investigation of the anatomy of the parts concerned in it has removed those difficulties, and dispelled those apprehensions. It may now be considered comparatively safe by the surgeon, who is acquainted with the works of Gimbernat and Hey, and the admirable production of Mr. Astley Cooper, and who is also practically versed in the anatomy of the parts. As this operation has not been practised here till lately, I have taken the liberty of laying a case of it before you, with the hope of its exciting attention to this subject.

Mrs. C. a lady residing fifty miles from Boston, had been affected with crural hernia sixteen years. On the 28th of January, 1807, from a slight cause, she was attacked with the symptoms of strangulation, which continued with incressing violence till the 4th of February, when I saw her. A small, quick, and weak pulse, tense abdomen, constant vomiting, and great tenderness of the diseased part, indicated that no time should be lost. Such efforts for the reduction by the taxis were made as the sensibility of the part allowed; and these proving ineffectual, the operation was decided on by us, and assented to by the patient.

The tumour was three inches long, and two wide; extending in the direction of the crural arch. Mrs. C. being placed on a table three feet and a half high, the legs hanging over the end of it, an incision, four inches long, was made across the hernia, at the termination of its inner third. This exposed the fascia, very thin, and requiring cautious dissection; after which the hernial sac appeared, and was opened at its lower part without appearance of water. When the sac was dilated, a dark coloured and apparently disorganized mass was seen; and it was not without frequent turning and examination, that it could be ascertained to be a ball of omentum enclosing a portion of intestine, which closely adhered together.—These were separated with some hazard; for the adhesion was so firm, the parts so absolutely of the same colour, and the intestine so closely invested on all sides by the omentum, that a momentary inadvertence might have allowed the intestine to be opened. Small portions of omentum were left on the intestine, rather than to incur an unnecessary risk in separating them. The intestine was then exam ned. Although it was very livid, yet as it possessed sensib lity fully sufficient to prove the existence of life, a a atte npt was was made to return it; but the stricture was much too narrow to suffer this to succeed. The obstruction, which first presented, was formed by the *falciform process* of Mr. Burns. When this was cut, the finger passed higher up to a stricture, produced by the internal edge of the crural arch, and the third insertion of the external oblique muscle. The tip only of the finger could be pushed into this stricture, by using a force somewhat greater than one would employ without its being indispensible. Then, the intestine being retained on the back of the finger, the curved bistory was insinuated on the fore part; the insertion of the external oblique cut toward the pubis; and, the knife being withdrawn, the finger passed into the abdomen. The intestine was then returned; but the omentum being enlarged, hardened, and conglomerated, a portion eight inches long was cut off, and a small ligature passed through the remainder, to retain the bleeding vessels at the mouth of the sac. Three ligatures were made through the skin, the wound was dressed with adhesive plaster, firm compresses, and bandage, and the patient laid in a state of ease and tranquillity, compared to that before the operation.

In two or three days some increased tension of the abdomen succeeded, and was relieved by the applications of the attending physician, Dr. S——. No other accidents ocurred, and Mrs. C. recovered a state of more perfect health than before; which she has since continued to enjoy.

### OBSERVATIONS ON THE TREATMENT OF HERNIA.

As the improved modes of practice, founded on the valuble observations of the celebrated Mr. Cline, and on those of Mr. Cooper and Mr. Hey, are not yet generally diffused, I beg the favour of being allowed to add to the above detail some remarks on the treatment of hernia.

The Spanish surgeon, Gimbernat, tells us, it has been computed, that an eighth part of the human species is affected with hernia. So little attention has been paid to this disorder, that it is difficult to form an opinion of the proportion in this country. It probably does not exceed one in fifty. Many suffer without suspecting it, especially females; numbers of whom, affected with a small, almost imperceptible tumour in the groin, are little aware that it constitutes a derangement, which if neglected, may produce a painful disease and death. It is here well known, that females, supposed to be affected with *bilious colic*, have have often concealed the existence of an abdominal tumour till too late, and perished miserably from a *strangulated hernia.*

The hernia, to which our attention is most commonly solicited, is the *inguinal hernia of males* in a state of strangulation. The treatment of it differs from that formerly employed. The old practice of raising the patient and shaking him by the heels, of using violent pressure to reduce the tumour, and of administering powerful cathartics, are now known to be unavailing, dangerous, and fatal. Correct anatomical and pathological views have given rise to the following methods.

1st. Let the patient affected with strangulated hernia be laid upon a bed, his head and pelvis a little raised. Let the feet be drawn toward the trunk, so as to elevate the knees, which should be in contact. The surgeon, standing on the side where the hernia lies, grasps it with one hand, makes a pressure toward the stricture, and with the thumb and a finger of the other hand moves from side to side the portion nearest to the abdominal ring. This process may be gently continued for half an hour.

2d. Should the TAXIS fail, it is advisable to take away blood in sufficient quantity to cause a general relaxation of muscular action, and, at the moment this happens, to renew the taxis. Blood letting may be repeated in co-operation with the other remedies.

3d. Before the muscles recover their action, apply to the hernia ICE, or if that be not procurable, ETHER, or COLD WATER.

4th. The INJECTION *of* TOBACCO is a powerful application. This should be used with some caution. Mr. COOPER relates a fatal case produced by the use of one drachm of tobacco. An empiric in this place, employing it more freely, destroyed his patient in fifteen minutes. The injection should be prepared by infusing a drachm of tobacco in a pound of boiling water for ten minutes. If the subject be of an irritable habit, half of this should be injected at first; but if the whole quantity do not produce symptoms of relaxation, another dose should be administered. This remedy produces distress, and appears to have the inconvenience of being often expelled, before it can act upon the system. When this has happened, the addition of eighty or one hundred drops of tincture of opium has been serviceable in effecting the retention.

5th. The WARM BATH has not perhaps been often successful after the injection of tobacco had entirely failed; but but it merits a trial. The temperature of the bath should be somewhat higher than that of the human body; and the space of time for bathing half an hour, which is long enough to afford all the good effects peculiar to the warm bath. The local application of cold may be conjoined.

6th. Opium is frequently a valuable emedy. It may be employed either merely to alleviate pain, or to effect a reduction. For the latter intention it is conveniently given, during the night, in repeated doses, while the other remedies are intermitted. In one case twenty grains taken in the course of a night produced a return of the hernia.

When these prove ineffectual, it does not seem advisable to exhaust the strength of the patient by other applications. The operation is now the only remaining resource, and to this recourse should be had, perhaps as soon as the others have been fairly tried; for although it may succeed at the end of a week, as in the case above related, yet I have seen it prove ineffectual at the end of forty-eight hours; and the disease has been known to be fatal in twenty-four hours.

After the reduction, the patient should be advised to put on a truss, and not to hazard the return of his complaint by relinquishing it within a short period. It has been usual to apply the truss upon the abdominal ring only; a practice founded on an erroneous idea of the egress of this hernia; for it does not issue from the abdomen opposite to that ring, but comes out through a fascia two inches above the ring, descends and piercing the tendon of the external oblique, appears at the external, or abdominal ring. The place therefore, where a truss should press in common cases, is at two inches, or an inch and a half, above the abdominal ring, toward the anterior superior spinous process of the ilium, or about midway between the os pubis and that process Mr. Cline first suspected the existence of hernia above the abdominal ring, as I think, from seeing a man perish with the symptoms of strangulated hernia without an external tumour. Mr. Cooper has dissected these parts, discovered the internal ring, and perfectly demonstrated this interesting part of anatomy.

